# International Congress on House Sanitation

**Published:** 1904-06-18

**Authors:** 


### INTERNATIONAL CONGRESS ON HOUSE SANITATION.

A CIRCULAR has just been issued by the "Société Française d'Hygiène" to announce that an International Congress on the sanitary improvement and salubrity of dwellings will be held under its auspices in Paris, from the 15th to the 20th of October, in connection with the *Exposition Internationale*, and that the collaboration of experts from all countries will be welcomed. The work of the Congress is to be divided among six sections, dealing respectively with town dwellings, rural dwellings, workmen's dwellings, hotels and furnished apartments, educational dwellings, and floating habitations, from battleships to canal barges. The aim of the Congress is declared to be to study the hygienic conditions of the construction or equipment of all places destined for habitation, to inquire into the improvements which may be introduced into their construction, management, or maintenance, and to ascertain the best methods of securing the application to them of the principles of hygiene, alike by municipalities, proprietors, shipowners, engineers, employers, and occupants. In each section a special report will be presented for discussion, having first been printed and distributed among the members, and communications from members will also be welcomed, but they must not occupy more than ten minutes in reading, and will not be discussed. Such communications must be received by the Secretary-General before September 1st. A volume of Transactions will be published, and will include the papers received from individual members, with certain reservations as to length, illustrations, and general character; papers which are definitely "commercial," or which do not fall within the scope of the Congress, being liable to rejection. Members of the Congress will pay a subscription of 20 francs, for which they will receive the right of admission to all the sectional meetings, with *un insigne artistique*, free admission to the Exposition during the continuance of the Congress, an invitation to all fêtes given on the occasion, a reduction of 50 per cent. on all railway fares in France, and the publications of the Congress. The wives of members will be invited to all fêtes and to the opening meeting, and may also take part in the meetings and excursions. Communications and applications for membership should be addressed to the Secretary-General of the Congress, M. F. Marié-Davy, 7 rue Brézin, Paris (14e Arrondt). Although the Congress is described as "International," there is no reference to any official language other than French, and the presidents and sectional officers mentioned are all French, many of them men of high distinction.

